# Rapid Identification and Verification of Indirubin-Containing Medicinal Plants

**DOI:** 10.1155/2015/484670

**Published:** 2015-05-18

**Authors:** Zhigang Hu, Yuan Tu, Ye Xia, Peipei Cheng, Wei Sun, Yuhua Shi, Licheng Guo, Haibo He, Chao Xiong, Shilin Chen, Xiuqiao Zhang

**Affiliations:** ^1^College of Pharmacy, Hubei University of Chinese Medicine, No. 1 Huangjiahu West Road, Hongshan District, Wuhan 430065, China; ^2^Institute of Chinese Materia Medica, China Academy of Chinese Medical Sciences, Beijing 100700, China

## Abstract

Indirubin, one of the key components of medicinal plants including *Isatis tinctoria, Polygonum tinctorium*, and *Strobilanthes cusia*, possesses great medicinal efficacy in the treatment of chronic myelocytic leukemia (CML). Due to misidentification and similar name, materials containing indirubin and their close relatives frequently fall prey to adulteration. In this study, we selected an internal transcribed spacer 2 (ITS2) for distinguishing these indirubin-containing species from five of their usual adulterants, after assessing identification efficiency of *matK, rbcL, psbA-trnH*, and ITS2 among these species. The results of genetic distances and neighbor-joining (NJ) phylogenetic tree indicated that ITS2 region is a powerful DNA barcode to accurately identify these indirubin-containing species and discriminate them from their adulterants. Additionally, high performance liquid chromatography (HPLC) was used to verify indirubin in different organs of the above species. The results showed that indirubin had been detected in the leaves of *Is. tinctoria, P. tinctorium, S. cusia*, and Indigo Naturalis (made from their mixture), but not in their roots, or in the leaves of their adulterants. Therefore, this study provides a novel and rapid method to identify and verify indirubin-containing medicinal plants for effective natural treatment of CML.

## 1. Introduction

**Figure 1:**
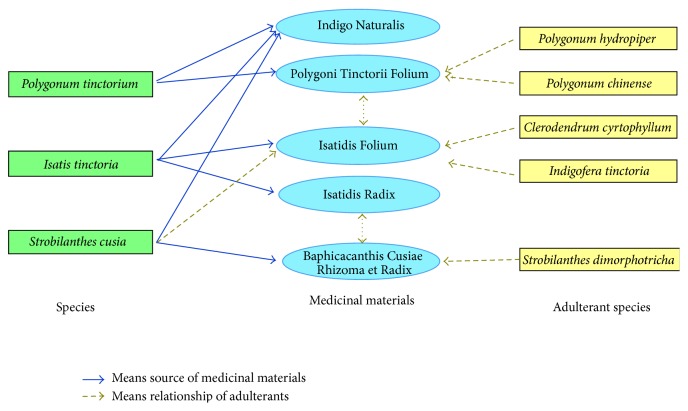
The relationship among indirubin-containing medicinal materials (plant organs/formulations), their plants of origin, and adulterants.

Chronic myelocytic leukemia (CML) is a malignant cancer that destroys the blood and marrow [1]. In 1967, a group of scientists discovered that the traditional Chinese medicine prescription, Danggui Luhui Wan, which contains 11 Chinese herbal medicines, had a significant curative effect on CML [2, 3]. More recently, indirubin from the Chinese herbal medicine Indigo Naturalis (Qingdai), prepared from the leaves of* Isatis tinctoria*,* Polygonum tinctorium,* and* Strobilanthes cusia*, was found to be the active ingredient [4–11]. Indirubin has since been found in additional Chinese herbal medicines derived from each of these species, respectively, including Isatidis Folium (the leaf of* Is. tinctoria*), Polygoni Tinctorii Folium (the leaf of* P. tinctorium*), and Baphicacanthis Cusiae Rhizoma et Radix (the root and rhizome of* S. cusia*) [12–14]. However, few studies have determined the presence and containing of indirubin in the above medicinal plants and their adulterants. These adulterants include* P. hydropiper, P. chinense, Clerodendrum cyrtophyllum, Indigofera tinctoria,* and* S. dimorphotricha* (Figure 1) and are extremely difficult to discriminate from their true medicinal counterparts morphologically [15–18]. The confusion between true medicinal plants and their adulterants has adverse effects on the clinical efficacy and safety of traditional medicines. It is thus paramount that traditional medicinal herbs and their active components can be reliably and cost-effectively discriminated from their false counterparts.

In order to achieve the rapid identification and verification of the indirubin-containing medicinal plants, two important problems must be resolved. First, the original plant species known to contain indirubin must be effectively discriminated from their adulterants. DNA barcoding technology offers the best technique for this to date, involving specific amplification of a short, standardized DNA fragment with universal primers across multiple samples [19, 20]. In recent years, several candidate DNA regions, such as* matK*,* rbcL*,* psbA-trnH,* and ITS/ITS2, have been assessed for their potential as DNA barcodes in plants [21–25]. Analysis of 50,790 plant ITS2 sequences revealed this region to be highly effective in discriminating medicinal plants and their closely related species [23, 26], more so than* matK*,* rbcL,* and* psbA-trnH*. As such, ITS sequences have been recommended as the core barcode region for seed plants [27]. Here, we reveal the first rapid and effective method to reliably discriminate indirubin-containing species in traditional medicinal formulations from their adulterants, using ITS2 barcoding. Secondly, it is necessary that the different plant organs comprising traditional medicinal materials (Figure 1) can be analysed for indirubin levels effectively. High performance liquid chromatography (HPLC) offers an effective screening technique in this regard.

## 2. Materials and Methods

### 2.1. Plant Materials

A total of 57 samples from 8 species were gathered from various geographical areas in China, as detailed in Table 1. All the specimens were carefully visually identified using standard expert identification parameters at the Institute of Medicinal Plant Development (IMPLAD), Chinese Academy of Medical Sciences. The specimens were deposited in the herbarium of the Hubei University of Chinese Medicine.

### 2.2. DNA Extraction, PCR Amplification, and Sequencing

Samples comprising 30~40 mg of dried leaves or 60~70 mg of roots were crushed into powder in 2 mL microfuge tubes at 30 Hz using stainless steel ball milling for 1 min. Total genomic DNA was extracted using a Plant Genomic DNA Kit (Tiangen Biotech Co., China) with modifications as follows. Initial incubation was at 65°C in 750 *μ*L GP1 (Tiangen) buffer for 1 h for dried leaves or 5 h for roots and rhizomes. The remaining steps followed the manufacturer's protocol.

For* matK*,* rbcL*,* psbA-trnH,* and ITS2 DNA barcodes, universal primers and general PCR reaction conditions were used as presented in Table S1 (see Table S1 in Supplementary Material available online at http://dx.doi.org/10.1155/2015/484670) [23, 28]. PCRs were in a 25 *μ*L reaction mixture, containing 30–100 ng of genomic DNA template, 12.5 *μ*L 2 × Tag PCR Master Mix (Aidlab Biotechnologies Co., China), and 1 *μ*L of forward and reverse primers (2.5 *μ*mol/L). After PCR, a 4 *μ*L aliquot was examined by 0.5% TBE agarose gel electrophoresis, and purified PCR products were sequenced in both directions using the primers used for PCR on an ABI3730XL sequencer (Applied Biosystems Co., USA).

### 2.3. Cloning and Sequencing of the ITS2 Region

The ITS2 PCR products of* S. cusia* and* S. dimorphotricha* were unsuccessfully directly sequenced. Therefore, purified products (TIANquick Midi Purification Kit; Tiangen Biotech Co., China) were ligated into the pMD18-T vector (Takara Biotech Co., China) and transformed into* E. coli* DH5 cells using standard recombinant DNA techniques. Positive transformants were selected on LB containing 0.1 mg/mL ampicillin and confirmed with colony PCR using the above PCR conditions. Four positive clones from each sample were sequenced on an ABI3730XL sequencer. In total, 40 clones from 13 samples of* S. cusia* and 7 clones from 3 samples of* S. dimorphotricha* (excluding fungal sequences) were obtained.

### 2.4. Sequence Analyses

Sequence editing and contig assembly were performed using CodonCode Aligner v4.25 (CodonCode Co., USA). The ITS2 region was obtained based on the HMMER annotation method to remove the 5.8S and 28S sections at both ends of the sequences [29]. Obtained DNA sequences were aligned and the intraspecific variation and interspecific divergence calculated by Kimura two-parameter method. Phylogenetic trees were constructed using the Neighbor-Joining method with molecular evolutionary genetics analysis (MEGA) software version 5.0 [30].

### 2.5. HPLC Analyses

The reference standard of indirubin was purchased from Shanghai Yuanye Bio-Technology Company (HPLC-tested purity >98%). 1.25 mg of indirubin was dissolved in N,N-dimethyl formamide in a 25 mL volumetric flask. Five milliliters of solution was transferred to another 50 mL volumetric flask containing N,N-dimethyl formamide to make a standard stock solution of 5 *μ*g/mL indirubin. Two-three replicate samples from each tested plant organ and species were randomly sampled for indirubin content. Specifically, 75 mg of powdered crude materials was sonicated in 10 mL of N,N-dimethyl formamide for 30 min at room temperature and filtered. A 20 *μ*L aliquot of the filtrate was applied to a HPLC column (Angilent TC-C_18_, 5.0 *μ*m, 4.6 mm × 250 mm). The optimum separation of HPLC was carried out with a mobile phase composed of methanol-water (75 : 25, v/v) at a flow-rate of 1 mL/min. Peaks were detected at a wavelength and column temperature of 290 nm and 25°C, respectively.

## 3. Results

### 3.1. Efficiency of Amplification and Identification for Four Candidate Barcodes

For all eight species tested, 24 samples were selected randomly for amplification efficiency. The efficiency of amplification of* matK, rbcL*,* psbA-trnH,* and ITS2 was 62.5%, 79.2%, 100%, and 100%, respectively. High-quality bidirectional sequences were obtained for all PCR products. All the GenBank No. were listed in Table S2 and Table 1. Alignment revealed no interspecific divergence between* S. cusia* and* S. dimorphotricha* using both* psbA-trnH* and* rbcL*. In addition,* matK* had low amplification efficiency (62.5%), making this less applicable for barcoding of these species. In comparison with the other barcodes, all 24 samples were successfully classified into eight species using the ITS2 sequence. Therefore, only the ITS2 barcode was used for further analysis.

### 3.2. Measurement of DNA Divergence for ITS2

Song et al. (2012) used sequence-tagged pyrosequencing and genome-wide analyses to describe intragenomic variations of ITS2 regions from 178 plant species. This study defined “major variants” as any variant whose relative variant abundance (RVA) was greater than 5% [31]. In this study we obtained 40 clones of* S. cusia* and only 2 of them (KJ939104, KJ939105) showed significant differences when compared with the other sequences. The remaining 38 sequences were considered major variants of ITS2 in this paper.

In this study, 86 sequences of ITS2 were obtained from all samples. Two ITS2 sequences (EU196919, JN235085) of* P. tinctorium* were downloaded from GenBank. The sequence length, GC average content, haplotype number and number of variable sites in each species (MEGA 5.0 software) are presented in Table 2. ITS2 sequence length ranged from 191 bp to 263 bp and GC average content ranged from 45.9% to 73.6%. The GC average contents of clones of* S. cusia* (73.6%) and* S. dimorphotricha* (73.0%) were at least 4% greater than those of the other species. Based on the variable sites,* Is. tinctoria, P. tinctorium,* and* S. cusia* were divided into 5, 1, and 15 haplotypes, respectively.

Interspecific and intraspecific distances using Kimura two-parameter method are shown in Table 3. The maximum intraspecific distances of* Is. tinctoria, P. tinctorium,* and* S. cusia* were 0.027, 0.000, and 0.036, respectively, while the minimum interspecific distance was 0.401. Furthermore, the minimum interspecific distances between any one of these three species and its adulterants were 0.514, 0.025, and 0.065, respectively.

### 3.3. Identification of ITS2 Using NJ Tree

**Figure 2:**
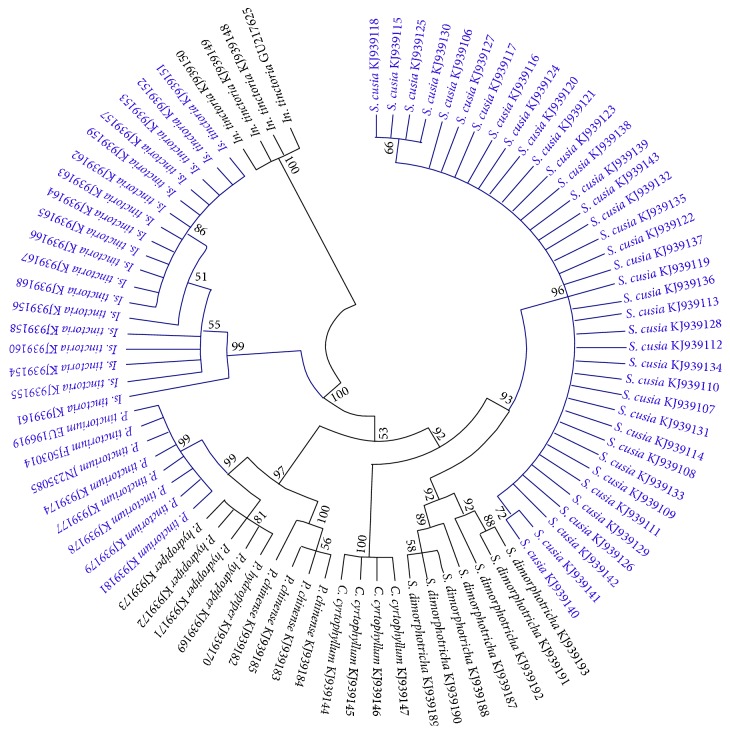
Phylogenetic tree of all the medicinal plants containing indirubin and their adulterants constructed with the ITS2 sequences using NJ method (Bootstrap scores ≥50%). The samples marked with blue represent the medicinal plants containing indirubin, and the others represent their adulterants.

Phylogenetic analysis demonstrated that every species clustered into their own clade, supported with at least 81% bootstrapping (Figure 2). In addition, three closely related species of the genus* Polygonum* were strongly supported (99%, 81% and 100% bootstrap, resp.), and clustered into a larger branch with 97% bootstrap. All cloned sequences of* S. cusia* (96% bootstrap) and* S. dimorphotricha* (92% bootstrap) in Acanthaceae family formed a larger group (93% bootstrap).

### 3.4. Detection of Indirubin in Crude Drugs and Their Adulterants

**Figure 3:**
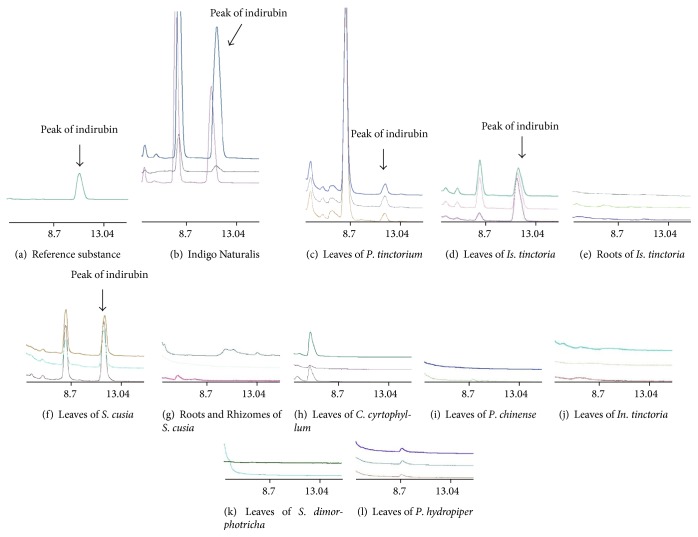
HPLC profiles of N,N-dimethyl formamide extract prepared from different organs of medicinal plants and five of their usual adulterants. Each profile was made up of two-three replicate samples from each tested plant organ and species.

HPLC detected indirubin in the leaves of* Is. tinctoria, P. tinctorium, S. cusia,* and Indigo Naturalis (Figure 3). Indirubin was not detected in the roots and rhizomes of these three taxa or in the leaves of their adulterants (*P. hydropiper, P. chinense, C. cyrtophyllum, In. tinctoria,* and* S. dimorphotricha*).

## 4. Discussion

In previous studies, the identification methods of medicinal plants including* Is. tinctoria*,* P. tinctorium,* and* S. cusia* have primarily focused on characterization of morphology, chromatographic fingerprints, and microstructures [32–34]. However, these methods all have their disadvantages. The recent, rapid development of DNA molecular marker techniques provides a powerful tool for the accurate identification of medicinal materials. In recent years, DNA barcoding has been successfully employed in species identification of medicinal herbs, with the ITS2 barcode exhibiting remarkable stability and accuracy in this field. ITS/ITS2 regions were demonstrated to successfully distinguish Corni Fructus (the flesh of* Cornus officinalis*) from its adulterants [35]. Xin et al. (2013) presented the ITS2 barcode as a powerful tool for tracing Goji (the fruit of* Lycium barbarum*) [36], while it was also used to accurately identify Ephedrae Herba (the stem taken from three species of* Ephedra*) and their closely related species [37]. Consequently, the rapidly developing DNA barcoding can effectively supplement the traditional identification methods. In this study, the ITS2 region was selected from four candidate barcodes to identify three species and their adulterants because of 100% amplification efficiency herein, high interspecific divergence, and low intraspecific variation. Based on the ITS2 barcode, the maximum intraspecific distance of the three species (*Is. tinctoria, P. tinctorium* and* S. cusia*) was less than the minimum interspecific distance, not only among the three species, but also among each species and its adulterants. Furthermore, the NJ tree indicated that* Is. tinctoria, P. tinctorium,* and* S. cusia* were clustered into their own monophyletic group, separated from the other species. Moreover, NJ tree analysis using ITS2 reliably distinguished individuals of the genus* Polygonum* and the family of Acanthaceae, supporting the powerful identification ability of ITS2 barcode in plants. Therefore, both the results of nearest distance method and NJ tree strongly support that ITS2 as DNA barcode can successfully distinguish* Is. tinctoria, P. tinctorium,* and* S. cusia* from each other and from their respective adulterants.

The demonstrated anticancer function of indirubin in the treatment of Chronic myelocytic leukemia (CML), warrants its further investigation and ability to be identified accurately in natural medicines. In this study, HPLC detection found that indirubin could only be detected in the leaves of* Is. tinctoria, P. tinctorium*,* S. cusia* and Indigo Naturalis. Meanwhile it could not be found in the root tissues of these species, or in the adulterant species tested herein. These results confirm that* Is. tinctoria, P. tinctorium,* and* S. cusia* cannot be replaced by their adulterants as indirubin-containing tinctures. And not only that, the species used for traditional medicinal herbs can be extremely disordered because of a general divergence in regional customs and species identification abilities [38]. All of these strongly supported the need for accurate discrimination of these ineffective false “pseudo”-medicines.

## 5. Conclusion

Together with HPLC detection of indirubin in various organs, ITS2 DNA barcoding enables the rapid, efficient, and cost-effective discrimination of the truly effective preparations of medicinal plants from their noneffective organs and adulterants that do not contain indirubin. This provides an efficient and new method to verify indirubin-containing medicines for the natural treatment of CML.

## Supplementary Material

The supplementary material contains Table S1 and Table S2. Table S1 showed the universal primers and reaction conditions of 4 candidate barcodes in this paper. Except for ITS2 barcode which was used for further analysis, the GenBank No. of matK, rbcL and psbA-trnH barcodes obtained from 24 samples of all eight species in this paper, were all listed in Table S2.

## Figures and Tables

**Table 1:** Detailed description of all the samples in this study.

Species	Medicinal part	Locality	Voucher number	GenBank number of ITS2
*Isatis tinctoria *	Leaves	Hebei, China	YC0021MT02	KJ939152
* Is. tinctoria *	Leaves	Anhui, China	YC0021MT09	KJ939157
* Is. tinctoria *	Leaves	Chongqing, China	YC0021MT10	KJ939158
* Is. tinctoria *	Leaves	Chongqing, China	YC0021MT12	KJ939159
* Is. tinctoria *	Leaves	Chongqing, China	YC0021MT13	KJ939160
* Is. tinctoria *	Leaves	Yunnan, China	YC0021MT14	KJ939161
* Is. tinctoria *	Leaves	Beijing, China	YC0021MT15	KJ939162
* Is. tinctoria *	Leaves	Beijing, China	YC0021MT04	KJ939154
* Is. tinctoria *	Leaves	Beijing, China	YC0021MT05	KJ939155
* Is. tinctoria *	Leaves	Beijing, China	YC0021MT06	KJ939156
* Is. tinctoria *	Roots	Sichuan, China	YC0021MT01	KJ939151
* Is. tinctoria *	Roots	Hebei, China	YC0021MT03	KJ939153
* Is. tinctoria *	Roots	Beijing, China	YC0021MT20	KJ939163
* Is. tinctoria *	Roots	Beijing, China	YC0021MT21	KJ939164
* Is. tinctoria *	Roots	Beijing, China	YC0021MT22	KJ939165
* Is. tinctoria *	Roots	Beijing, China	YC0021MT23	KJ939166
* Is. tinctoria *	Roots	Sichuan, China	YC0021MT29	KJ939167
* Is. tinctoria *	Roots	Hubei, China	YC0021MT30	KJ939168
*Polygonum tinctorium *	Leaves	Beijing, China	YC0390MT04	KJ939177
*P. tinctorium *	Leaves	Beijing, China	YC0390MT05	KJ939178
*P. tinctorium *	Leaves	Beijing, China	YC0390MT01	KJ939174
*P. tinctorium *	Leaves	Beijing, China	YC0390MT07	KJ939179
*P. tinctorium *	Leaves	Beijing, China	YC0390MT09	KJ939181
*P. tinctorium *	Leaves	Fujian, China	PS2901MT01	FJ503014
*Strobilanthes cusia *	Leaves	Guangdong, China	YC0389MT01	KJ939116-KJ939119
*S. cusia *	Leaves	Chongqing, China	YC0389MT02	KJ939109-KJ939112
*S. cusia *	Leaves	Chongqing, China	YC0389MT03	KJ939113-KJ939115
*S. cusia *	Leaves	Hainan, China	YC0389MT04	KJ939125-KJ939127, KJ939104
*S. cusia *	Leaves	Yunnan, China	YC0389MT07	KJ939139, KJ939140
*S. cusia *	Leaves	Yunnan, China	YC0389MT08	KJ939141-KJ939143
*S. cusia *	Leaves	Guangxi, China	YC0389MT10	KJ939105, KJ939137, KJ939138
*S. cusia *	Leaves	Fujian, China	YC0389MT11	KJ939133-KJ939136
*S. cusia *	Leaves	Guangxi, China	YC0389MT12	KJ939120-KJ939122
*S. cusia *	Leaves	Guangxi, China	YC0389MT13	KJ939123, KJ939124
*S. cusia *	Roots and rhizomes	Hainan, China	YC0389MT05	KJ939128-KJ939130
*S. cusia *	Roots and rhizomes	Hainan, China	YC0389MT06	KJ939131, KJ939132
*S. cusia *	Roots and rhizomes	Guangdong, China	YC0389MT14	KJ939106-KJ939108
*Polygonum hydropiper *	Leaves	Guangdong, China	YC0509MT01	KJ939169
*P. hydropiper *	Leaves	Guangdong, China	YC0509MT02	KJ939170
*P. hydropiper *	Leaves	Guangdong, China	YC0509MT03	KJ939171
*P. hydropiper *	Leaves	Guangdong, China	YC0509MT04	KJ939172
*P. hydropiper *	Leaves	Guangdong, China	YC0509MT05	KJ939173
*Polygonum chinense *	Leaves	Guangxi, China	YC0510MT01	KJ939182
*P. chinense *	Leaves	Guangxi, China	YC0510MT02	KJ939183
*P. chinense *	Leaves	Guangdong, China	YC0510MT03	KJ939184
*P. chinense *	Leaves	Guangdong, China	YC0510MT04	KJ939185
*Clerodendrum cyrtophyllum *	Leaves	Guangxi, China	YC0508MT01	KJ939144
*C. cyrtophyllum *	Leaves	Guangxi, China	YC0508MT02	KJ939145
*C. cyrtophyllum *	Leaves	Guangxi, China	YC0508MT03	KJ939146
*C. cyrtophyllum *	Leaves	Guangxi, China	YC0508MT04	KJ939147
*Strobilanthes dimorphotricha *	Leaves	Guangdong, China	YC0511MT01	KJ939187, KJ939188, KJ939191
*S. dimorphotricha *	Leaves	Guangdong, China	YC0511MT02	KJ939189, KJ939190
*S. dimorphotricha *	Leaves	Guizhou, China	YC0511MT03	KJ939192, KJ939193
*Indigofera tinctoria *	Leaves	Guangxi, China	YC0707MT01	KJ939148
*In. tinctoria *	Leaves	Guangxi, China	YC0707MT02	KJ939149
*In. tinctoria *	Leaves	Guangxi, China	YC0707MT03	KJ939150
*In. tinctoria *	Leaves	Guangxi, China	PS0251MT02	GU217625

**Table 2:** Sequence characteristics of the related species.

Species/(number of sequences)	Length of ITS2 (bp)	GC average content (%)	Number of haplotypes	Number of variable sites
*Is. tinctoria *(18)	191	56.7	5	5
*P. tinctorium *(8)	245	68.2	1	1
*S. cusia *(38)	230~235	73.6	15	20
*P. hydropiper *(5)	244	68.9	1	1
*P. chinense *(4)	263	65.8	1	1
*C. cyrtophyllum *(4)	224	56.7	1	1
*S. dimorphotricha *(7)	224~233	73.0	6	13
*In. tinctoria *(4)	219	45.9	1	1

**Table 3:** Data of interspecific and intraspecific distances of the related species.

Parameter	Range
Intraspecific distances of* Is. tinctoria *	0.000~0.027
Intraspecific distances of *P. tinctorium *	0.000
Intraspecific distances of *S. cusia *	0.000~0.036
Interspecific distance among the above three species	0.401~0.684
Interspecific distance between* Is. tinctoria* and its adulterants	0.514~0.684
Interspecific distance between* P. tinctorium* and its adulterants	0.025~0.755
Interspecific distance between *S. cusia* and its adulterants	0.065~0.931
