# Supplementary material for: Rapid Identification and Verification of Indirubin-Containing Medicinal Plants
Source: Evid Based Complement Alternat Med. 2015 May 18;2015:484670. doi: 10.1155/2015/484670 (PMC4451998; doi:10.1155/2015/484670)
Supplement: Supplementary file 1 — The supplementary material contains Table S1 and Table S2. Table S1 showed the universal primers and reaction conditions of 4 candidate barcodes in this paper. Except for ITS2 barcode which was used for further analysis, the GenBank No. of matK, rbcL and psbA-trnH barcodes obtained from 24 samples of all eight species in this paper, were all listed in Table S2. [file 484670.f1.pdf]

Supplementary Materials

TABLE S1: List of universal primers and reaction conditions for candidate barcodes.

DNA Barcode	Name of primers	Primer sequences 5′-3′	PCR reaction conditions
matK	KIM_3F<br>KIM_1R	CGTACAGTACTTTTGTGTTTACGAG<br>ACCCAGTCCATCTGGAAATCTTGGTTC	94 ℃ 1 min<br>94 ℃ 30 s, 52 ℃ 20 s, 72 ℃ 50 s, 35 cycles<br>72 ℃ 5 min
rbcL	1f<br>724r	ATGTCACCACAAACAGAAAC<br>TCGCATGTACCTGCAGTAGC	95 ℃ 2 min<br>94 ℃ 1 min, 55 ℃ 30 s, 72 ℃ 1 min, 34 cycles<br>72 ℃ 7 min
psbA-trnH	fwd PA<br>rev TH	GTTATGCATGAACGTAATGCTC<br>CGCGCATGGTGGATTCACAATCC	94 ℃ 5 min<br>94 ℃ 1 min, 55 ℃ 1 min, 72 ℃ 1.5 min, 30 cycles<br>72 ℃ 7 min
**ITS2**	S2F<br>S3R	ATGCGATACTTGGTGTGAAT<br>GACGCTTCTCCAGACTACAAT	94 ℃ 5 min<br>94 ℃ 30 s, 56 ℃ 30 s, 72 ℃ 45 s, 40 cycles<br>72 ℃ 10 min

TABLE S2: List of the GenBank No. for *matK*, *rbcL* and *psbA-trnH* barcodes (amplification and sequencing successfully).

Species	Voucher No.	GenBank No.		
		matK	*rbcL*	*psbA-trnH*
Isatis tinctoria	YC0021MT29	KJ939231	KJ939241	KJ939205
Is. tinctoria	YC0021MT05	KJ939229	KJ939240	KJ939203
Is. tinctoria	YC0021MT20	KJ939230	KJ939242	KJ939204
Polygonum tinctorium	YC0390MT01	KJ939218	KJ939248	KJ939214
P. tinctorium	YC0390MT04	KJ939219	KJ939247	KJ939212
P. tinctorium	YC0390MT05	KJ939220	KJ939246	KJ939213
Strobilanthes cusia	YC0389MT07	KJ939223	KJ939233	KJ939194
S. cusia	YC0389MT01	KJ939221	KJ939234	KJ939196
S. cusia	YC0389MT04	KJ939222	KJ939232	KJ939195
Polygonum hydropiper	YC0509MT02	-	KJ939243	KJ939209
P. hydropiper	YC0509MT03	-	KJ939244	KJ939210
P. hydropiper	YC0509MT04	-	KJ939245	KJ939211
Polygonum chinense	YC0510MT01	-	-	KJ939208
P. chinense	YC0510MT02	-	-	KJ939207
P. chinense	YC0510MT03	-	-	KJ939206
Clerodendrum cyrtophyllum	YC0508MT01	KJ888428	KJ939236	KJ939197
C. cyrtophyllum	YC0508MT02	KJ939224	KJ939237	KJ939198
C. cyrtophyllum	YC0508MT03	KJ939225	KJ939235	KJ939199
Strobilanthes dimorphotricha	YC0511MT01	-	KJ939249	KJ939215
S. dimorphotricha	YC0511MT02	-	KJ939250	KJ939216
S. dimorphotricha	YC0511MT03	-	-	KJ939217
Indigofera tinctoria	YC0707MT01	KJ939226	-	KJ939202
In. tinctoria	YC0707MT02	KJ939227	KJ939238	KJ939200
In. tinctoria	YC0707MT03	KJ939228	KJ939239	KJ939201
